# Identification and formation mechanism of key elements of supply chain resilience: Exploration based on grounded theory and verification of SEM

**DOI:** 10.1371/journal.pone.0293741

**Published:** 2023-11-02

**Authors:** Youan Ke, Lin Lu, Xiaochun Luo

**Affiliations:** 1 School of Economics and Management, Guangxi Normal University, Guilin, China; 2 School of Economics and Management, Nanjing University of Aeronautics and Astronautics, Nanjing, China; King Khalid University, SAUDI ARABIA

## Abstract

Supply chain resilience has garnered significant attention from both scholars and practitioners. However, the complex nature of the topic has resulted in a dearth of research on its key elements and formation mechanisms. To bridge this knowledge gap, we implemented grounded theory and conducted semi-structured interviews with 23 interviewees, which led to the identification of six key elements of supply chain resilience through open coding, axial coding, selective coding, and theoretical model saturation testing. These elements are product supply resilience, resource resilience, partner resilience, information response resilience, capital resilience, and knowledge resilience. Drawing from the key elements and the three phases of supply chain resilience (readiness, response, and recovery), we illustrated its formation mechanism and constructed a theoretical model of the influencing factors and pathways of supply chain resilience. We devised a questionnaire based on the coding results and confirmed its reasonableness and validity with a small sample of 109 questionnaires. Subsequently, a large sample of 409 questionnaires was used to test and validate the theoretical model using structural equation modeling, demonstrating that the identified key elements positively impact supply chain resilience. In sum, our paper enriches the comprehension of supply chain resilience by identifying its key elements and elaborating on its formation mechanism.

## 1. Introduction

In the context of economic globalization, labor specialization, digital intelligence, and the upgrading of personalized customer needs, supply chain networks are gradually evolving into supply chain ecosystems, resulting in a more complex structure that increases the likelihood that it will be affected by unexpected events [1]. Unfortunately, statistics indicate that nearly 75% of organizations experience supply chain disruptions annually [2], and these disruptions caused by risk can be expensive for organizations. Two distinct types of risks frequently impact supply chains: operational risk, which is related to systematic supply chain business risks and is due to the disturbance caused by the uncertainty of day-to-day operations, such as supply delays, demand fluctuations, equipment failures, and product quality. The other type of risk is disruption risk, caused by unexpected events that significantly impact the supply chain, such as natural disasters, wars, and policy changes. This type of risk is difficult to predict, has a low likelihood but a high impact, and may have irreversible negative consequences [3, 4]. Both types of risk, which are essentially the result of unpredictable internal and uncontrollable external factors, have the potential to disrupt the supply chain, and the latter type is more likely to do so.

Supply chain disruption is characterized by urgency, uncertainty, unpredictability, destructiveness, and diversity, which impede the receipt and delivery of expected goods and limit the ability of node companies to provide services to customers [5]. These impact the operational efficiency, market share, and profitability of supply chain nodes, ultimately weakening the performance of the supply chain through decreased revenue, customer complaints, lower productivity, and higher production costs. A more resilient supply chain can reduce the destructiveness and duration of supply chain disruption more effectively than its competitors and recover from disruption rapidly, thereby mitigating the impact of disruption on supply chain performance. Identifying the key elements of supply chain resilience, and comprehending and enhancing supply chain resilience, have significant implications for the future of nations and organizations [6].

Scholars have conducted extensive research on the elements of supply chain resilience, and some have worked on research projects related to the key elements of supply chain resilience [7–11]. However, few have focused their efforts on identifying the key elements. Understanding and enhancing supply chain resilience focuses on identifying its key elements and formation mechanism, enabling companies to invest efficiently with limited resources and effectively enhance supply chain resilience. Given the importance of supply chain resilience, the numerous elements affecting supply chain resilience, and the paucity of research on the identification of key resilience elements, this study employs grounded theory to conduct an exploratory study of key elements and the formation mechanism of supply chain resilience to answer two questions:

RQ1: Among the various elements of supply chain resilience, what are the key elements?
RQ2: At what phases of supply chain resilience do these key elements become operational, and what is the formation mechanism of supply chain resilience?

The following are the prospective innovations of this study: First, it enriches the study of key elements of supply chain resilience based on the dearth of scholarly research, and then it constructs and evaluates a scale for key elements of supply chain resilience. Second, this study innovatively integrates the key elements into the capabilities and three phases of supply chain resilience and uses a combination of qualitative and quantitative methods to construct a theoretical analysis framework of supply chain resilience based on the key elements, thereby enhancing the academic community’s understanding of the key elements and formation mechanism of supply chain resilience.

## 2. Literature review

### 2.1. Concepts of supply chain resilience

The demand for supply chain resilience is based on the fundamental premise that none of the risks can be eliminated entirely [12]. Organizations may lessen the disruption risks to the continued functioning of the supply chain by enhancing resilience, which is the prerequisite of the demand for resilience [13]. At the same time, improving supply chain resilience will surely be accompanied by a large amount of resource investment, which will inevitably increase the resilience shaping cost, and the excessive cost is bound to erode the profits of enterprises [14, 15]. In an environment that is dynamic and unpredictable, the ideal and actual states often diverge. Any supply chain disruption can have a ripple effect, with local disruptions propagating along the supply chain network and causing disruptions in the entire supply chain [16, 17]. In order to avoid disruptive effects, the supply chain must function even when disruptions occur. An emphasis on supply chain resilience has arisen due to the significant losses resulting from supply chain disruptions and their increasing frequency. Supply chain resilience is critical for preventing and responding to supply chain disruptions and ensuring continuity of supply chain operations. Therefore, supply chains need to be resilient to cope with the effects of environmental change.

According to Christopher & Peck [18], supply chain resilience refers to the capability of a system to bounce back to its original or more desirable state after the disturbance. Williams et al. [19] consider supply chain resilience as the ability of a supply chain to respond to unexpected disruptions and resume normal supply chain network operations. Dubey et al. [20] define supply chain resilience as the capability of a supply system to recover to its original state within an acceptable period after a disruption. It is clear from these definitions above that supply chain resilience is only defined in terms of post-response, which is reactive. Supply chain resilience can introduce the concept of disruption prevention to reduce the likelihood of disruptions and avoid their effects. According to Falasca et al. [21], supply chain resilience is the capability of a supply chain to reduce the likelihood of disruption, mitigate the consequences of disruption, and shorten the time it takes for the system to resume regular operation. Ponomarov and Holcomb [22] consider supply chain resilience as the adaptive capacity to cope with unforeseen events, respond to disruptions and recover from them. Hohenstein et al. [7] argue that supply chain resilience is the supply chain’s capability to be prepared for unexpected risk events, responding and recovering quickly to potential disruptions to return to its original situation or grow by moving to a new, more desirable state, which somehow affirms the supply chain’s capability to learn after disruptions and to turn threats into opportunities. Supply chain resilience is the capability of a supply chain to respond to disruptive events through both proactive and reactive forms [23].

A review of definitions reveals no consensus regarding the definition of supply chain resilience but that academics have consistently developed a comprehensive knowledge of the concept. It has evolved from reactive responses after the disruption to include both proactive preventions before disruption and reactive responses after the disruption. The capability to learn allows the supply chain to recover and function at a higher level, which is another aspect of supply chain resilience. In addition, supply chain resilience is confused with terms such as disruption, disturbance, disruptive event, and unexpected event, and there is no consensus. Particularly, disruption, disruptive events, and unexpected events narrow the set of possible events [24]. Disturbances are foreseen or unforeseen events that directly affect the regular operation and stability of an organization or supply chain [25] and are the pre-process and the manifestation of supply chain disruption. The supply chain will function normally if resilience successfully absorbs the disturbances. Otherwise, disruptions will occur, and supply chain disruption is the sequential result of the disturbance [26]. In this study, therefore, the term disturbance is used, which encompasses a broader range of events and more accurately depicts the reality of supply chain conditions prior to disruptions. In addition to rapid and efficient responses to disruptions, supply chain resilience requires monitoring and identifying risks to prevent disruptions. Many academicians view supply chain resilience as a capability that encompasses the capability to absorb, adapt, and recover [27–29]. In conclusion, this study defines supply chain resilience as a capability that includes the capability to absorb disturbance events before a supply chain disruption, the capability to adapt to the environment by responding rapidly after the disruption, and the capability to recover the supply chain to its original level or even higher.

### 2.2. Characteristics of supply chain resilience

#### 2.2.1. Dynamic evolutionary

The supply chain system has a close relationship with the ecosystem. According to the contingency theory, for organizations to achieve a higher performance level, their elements and behaviors must be compatible with the environment. Organizations can maintain resilience in dynamic and complex environments by employing a diverse and dynamic multitude of practices [30]. Supply chain resilience is a dynamic concept [31]; the nature of supply chain resilience is that it changes with the environment, and environmental changes create new resilience requirements [32]. In the process of continuous environmental change, the supply chain requires a dynamic capability to match it so that it has the appropriate anti-risk capability in the three phases of resilience (readiness, response, and recovery); otherwise, supply chain disruptions will occur. In the context of environmental uncertainty, supply chain resilience can continuously integrate, construct, and reconfigure supply chain resources to match the specific environment and accomplish dynamic evolution in response to dynamic changes and the condition of the supply chain. Supply chain resilience is a dynamic evolutionary capability of the supply chain [33].

#### 2.2.2. Complexity

The supply chain is becoming more complex due to increased market competition, demand volatility and product portfolios, shorter product life cycles, and faster innovation rates [34, 35]. In addition, as each level of the supply chain expands, it progressively develops into a supply chain ecosystem with a growing number of nodes. The same supply chain node companies may also exist in other chains, which may interact. Given the necessity to deal with the increasing complexity of supply systems, academicians and practitioners have focused on supply chain resilience [36]. The dynamic evolution characterizes the supply chain’s adaptability to a dynamic environment by exhibiting diverse resilience conditions and initiatives in response to changing environments and at different phases of the supply chain resilience [37]. Supply chain resilience is complicated by the interaction of the two dimensions of supply chain management complexity and management environment complexity, which are related to the supply chain’s internal complexity and disrupted by the external environment’s dynamic complexity [38]. Consequently, complexity is a characteristic of supply chain resilience.

#### 2.2.3. Ambidexterity

According to prior research, supply chain resilience requires proactive and reactive measures to effectively address disruptions [39–41]. At the same time, the supply chain has been continuously extended at all levels, and is gradually transforming into a supply chain ecosystem with a gradual increase in the number of nodes involved. The node enterprises in the same supply chain may also be in different supply chains, and each supply chain interacts with each other. The supply chain is increasingly characterized as a complex social system, with deepening complexity of association with the social environment, dynamics and non-linearity. The need to address escalating supply chain complexity has generated interest in supply chain resilience among scholars and supply chain practitioners [42]. Both supply chains and supply chain resilience have their unique characteristics and are evolutionary responses to specific environments. The dynamic evolutionary nature of supply chain resilience characterizes the ability of supply chains to adapt to dynamic environments, exhibiting different resilience profiles and initiatives in the face of different environments as well as at different stages of the supply chain [43], and is the ability to adapt to the dynamic complexity of the environment. Supply chain resilience is both related to the internal complexity of the supply chain and disturbed by the dynamic complexity of the external environment, i.e., the two dimensions of supply chain management complexity and management environment complexity together contribute to the complexity of supply chain resilience [39]. Therefore, complexity characterizes supply chain resilience.

#### 2.2.4. Phase

**Fig 1:**
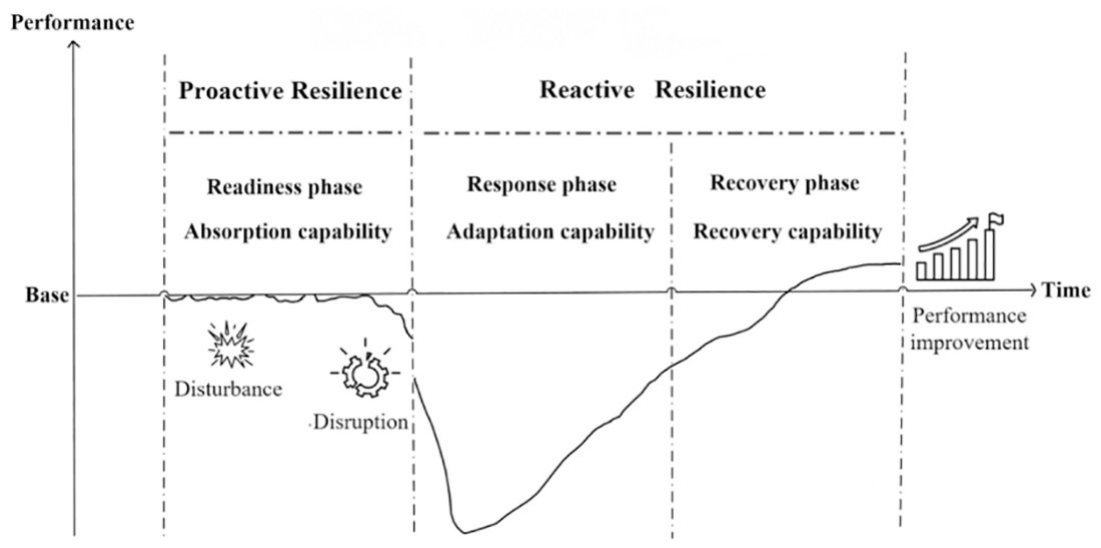
Three phases of supply chain resilience.

Supply chain resilience has been classified into different phases, each reflecting the peculiarities of the phase. The phases of supply chain resilience are readiness, response, and recovery [22, 23, 44], but Hohenstein et al. [7] argue that supply chain resilience consists of four phases: readiness, response, recovery, and growth, and the growth phase indicates that the supply chain can learn from disruptions and drive supply chain performance to a higher level. A more advanced manifestation of supply chain resilience is learning from disruptions and growing [45]. Knowledge management influences the recovery phase of supply chain resilience, which implies the supply chain’s capability to continuously learn from the feedback of disruptions to develop better plans and solutions for the future. Therefore, this study concurs with Ponomarov and Holcomb [22] that supply chain resilience includes three phases: readiness, response, and recovery, with the recovery phase also accounting for the growth implications. The three phases of supply chain resilience are depicted in Fig 1.

It is essential to recognize the possibility of disruption from any source and adequately prepare [46]. The readiness phase includes the forecasting, identification, and evaluation of environmental disturbances and the preparation for absorbing the disturbances to reduce the likelihood of disruption. The level of readiness is determined by how well the supply chain absorbs the disturbance. Following the failure of supply chain absorption disturbance, the response phase demonstrates a rapid response to supply chain disruptions as well as the dynamic evolutionary capability of resilience to continuously integrate, construct, and reconfigure the supply chain’s resources and capabilities in order to minimize disruption losses and prepare for subsequent recovery. The objective of the recovery phase is to quickly return the supply chain to its original state or even develop to a higher state while recovering and learning from the disruption.

### 2.3. Elements of supply chain resilience

According to academic research, recognizing the key elements of supply chain resilience requires clarifying the elements of supply chain resilience. Scholars have utilized inconsistent terminology when referring to supply chain resilience elements, these are referred to as enhancers [37], competencies [47], principles [25], elements [48], capabilities [28], antecedents [49], and enablers [50]. This study employs the term element, contributing to resilience development [51].

#### 2.3.1. Classical literature review sorting

This subsection reviews some of the scholars’ studies that identify the elements of supply chain resilience based on the systematic literature review approach, as shown in Table 1. In dynamic and complex environments, firms need to have the ability to perceive environmental uncertainty, and the possibility of supply chain disruptions can be reduced through proactive strategies such as increased awareness of environmental threats. In practice, when supply chain disruptions do not affect production, the probability is that they cannot attract management’s attention, and the lag in response strategies can cause irreparable harm to the supply chain [52]. From the results of literature review combing, scholars focus on certain elements of active resilience, such as supply chain risk management culture, situational awareness, and redundancy; at the same time, flexibility, redundancy, synergy, and agility are the focus of researchers’ attention, which deserves to be focused on; researchers usually add or adjust the elements that they think are needed on the basis of one another’s research; in addition to this, the elements of supply chain resilience, although becoming more and more comprehensive, but are somewhat cumbersome.

**Table 1:** Elements of supply chain resilience.

Scholar	Time span	Number of article	Element
Hohenstein et al. [7] (2015)	2003–2013	67	Flexibility, Redundancy, Collaboration, Visibility, Agility, Multiple sourcing, Capacity, Culture, Inventory, Information sharing, Human resource management, Predefined contingency plans
Kamalahmadi et al. [25] (2016)	2000–2015	100	Supply chain reengineering, Collaboration, Agility, Trust, Redundancy, Information sharing, Innovation, Supply chain risk management culture, Visibility, Velocity, Leadership,
Stone and Rahimifard [53] (2018)	2003–2016	137	Flexibility, Risk aware culture, Redundancy, Early warning detection systems, Security, Efficiency, Contingency plans, Financial strength, Leadership commitment, Relationships, Human resource management, Business continuity, Knowledge management, Market position, Robustness, Adaptive management, Collaboration, Agility, Visibility, Adaptability, Node criticality, Information flow, Velocity, Redundancy, Self-organisation, Rapidity, Established communication lines, Risk management orientation, Diversity, Network complexity, Cohesion, Co-Learning, Community resources, Bargaining power, Responsiveness, Buffer capacity
Ali and Gölgeci [49] (2019)	2003–2018	155	Flexibility, Redundancy, Collaboration, Resilience culture, Information sharing, Supply chain Innovation, Top management support, Employees training and development, Public–private partnership, Co-opetition, Industry 4.0, Big data analytics, Blockchain technology, Visibility, Robustness, Agility, Velocity, Resource reconfiguration, Adaptation, Disruption mitigation, Supply chain redesign, Additive manufacturing
Naimi et al. [8] (2022)	2009–2019	94	Flexibility, Redundancy, Trust, Information Sharing, Collaboration, Visibility, Velocity, Risk management culture, Supply chain reengineering, Multiple sourcing, Agility, Robustness, Adaptive capability, Management process, Backup capability, A goverment aid and program, Financial Strength, Risk and revenue sharing, Supply chain structure, Company knowledge, Sustainability in supply chain, Market sensitiveness, Readiness, response and recovery, Coordination and control, Lead time, Variability, Dynamic capabilities, Stockpiling Inventory, Changing climate, Security, Efficiency, Dispersion, Anticipation, Egocentric network-based strategies, Procurement portfolio, sensing, Seizing and transforming, Learning orientations

#### 2.3.2. Stage division combing

Numerous elements of supply chain resilience have been identified, and scholars have begun to explore which elements play a role in which stage of supply chain resilience, integrating the influencing elements of resilience into the stages of resilience. This is critical from a supply chain management perspective [11]. Table 2 summarizes the division of elements into different stages of supply chain resilience by some scholars, from which it can be observed that different scholars have different perspectives and identify different elements of resilience based on the differences in the division of resilience stages. It is worth noting that although the reference value is provided, the stage division of the elements is mostly summarized in the previous theoretical studies and lacks the support of empirical studies, which is insufficient in terms of scientificity and rigor. Therefore, there is a need to further clarify the stage division of supply chain resilience and the roles of numerous elements in each stage.

**Table 2:** Phases of supply chain resilience and influencing elements.

Scholar	Phases of supply chain resilience	Element
Hohenstein et al. [7] (2015)	Readiness	Collaboration, Human resource management, Inventory management, Predefined decision plans, Redundancy, Visibility
	Response	Collaboration, Flexibility, Human resource management, Redundancy
	Recovery	
	Growth	
Ali et al. [48] (2017)	Anticipate	Situation awareness, Robustness, Visibility, Security, Knowledge management (Pre-disruption)
	Adapt	Flexibility, Redundancy
	Respond	Collaboration, Agility
	Recover	Contingency planning, Market position
	Learn	Knowledge management (Post-disruption), social capital
Kochan and Nowicki [28] (2018)	Readiness	Efficiency, Dispersion, Market position, Security, Collaboration, Financial Strength, Revenue management, Market strength, Organizational culture, Anticipation
	Response	Agility, Redundancy
	Recovery	Adaptability, Crises management, Communication strategies, Resource mobilization, Consequence mitigation
Han et al. [45] (2020)	Readiness	Situation awareness, Visibility, Security, Redundancy
	Response	Agility, Flexibility, Collaboration, Leadership
	Recovery	Knowledge management, Contingency planning, Market position

### 2.4. The need to identify key elements of supply chain resilience

Based on the resource base theory, firms possess different tangible and intangible resources, and by integrating and optimizing these resources, they gain and sustain firm performance and competitive advantage [54]. For supply chains, firms improve supply chain resilience based on the resources they possess through initiatives such as redundancy, optimizing supply chain network design, and investing in IT infrastructure, which in turn positively affects the level of supply chain performance. However, it is important to note that firms have limited resources and it is unrealistic to invest in all aspects of resilience. Given the complexity of the elements of resilience, enterprises need to focus on the key elements of resilience, transform the key elements into the resources they possess, improve output efficiency and the value of resources, and build supply chain resilience effectively and efficiently.

Based on the dynamic capability theory, dynamic capability is an ability to continuously integrate, construct and reconfigure internal and external resources and capabilities to adapt to environmental changes and gain competitive advantages [55]. As mentioned earlier, supply chain resilience is a dynamic capability with dynamic evolutionary and stage characteristics, and its evolutionary nature is closely linked to environmental changes [31]. In the preparation, response and recovery phases of supply chain resilience, the enterprise’s own state and the environment in which it operates change, and enterprise decision-making is always confronted with a lot of uncertainty and complexity, and there is variability in the resources and capabilities required to build absorption, adaptation and recovery capabilities. Ideally, firms would be able to continuously adjust their resources and capabilities to maintain a dynamic match with the needs of the environment. However, within the constraints of limited enterprise resources and capabilities, it is more realistic for enterprises to develop key dynamic resilience capabilities with the resources and capabilities they have available.

## 3. Research design

### 3.1. Research methods

Grounded theory is a qualitative research method that combines empirical and theoretical research, proposed by American scholars Glaser and Strauss in 1967. It bridges the gap between traditional qualitative and quantitative research to a certain extent and is one of the more scientific qualitative research methods. The three primary schools of grounded theory are classical grounded theory, represented by Glaser; proceduralised grounded theory, represented by Strauss; and constructivist grounded theory, represented by Charmaz [56]. The three primary schools of grounded theory embody positivist, interpretivism, and constructivist epistemologies. The differences in epistemologies are reflected in their methodological differences, which lead to differences in their approaches, concentrated in the coding sessions.

Different research questions should be matched with different research methods, and this paper’s grounded theory research method has its rationale. Firstly, grounded theory is appropriate for studies with insufficient explanations or imperfect theory construction. Given the dearth of research on the key elements of supply chain resilience, it is reasonable to use grounded theory to explore the research. Secondly, it is based on research scenarios. Theoretical questions in management research should be derived from specific management situations [57], and grounded theory is essentially a bottom-up approach to theory construction through a set of systematic operations [58].

This study employs proceduralised grounded theory to identify key elements of supply chain resilience, which are then coded using the qualitative data analysis software NVivo20. The proceduralised grounded theory divides the coding procedure into three steps: open, axial, and selective. Open coding extracts initial concepts from the data and categorizes them; axial coding integrates the categories and refines the main categories by delving deeper into the true meaning of the initial concepts and categories and the logical relationships between them; and selective coding converges the core categories, explores the relationships between the main categories and the core categories, and creates a narrative between the main categories and the core categories. After coding, a theoretical saturation test is required to ensure the scientific and rigorous character of the theoretical model construction.

### 3.2. Data collection

#### 3.2.1. Interviewees

The data required for the grounded theory method can be derived from first-hand data such as research interviews or second-hand data such as research literature, policy documents, and news reports. In this investigation, semi-structured interviews are used to collect first-hand data. In order to enhance the quality of the interviews, it is also necessary to establish the criteria for selecting interviewees. The basic requirements for the selection of interviewees are as follows: (1) select employees with a bachelor’s degree or higher as interviewees to ensure a good performance in logical analysis, understanding and judgment, and presentation skills; (2) require interviewees to have more than one year experience in work related to the supply chain and be familiar with the operational processes of the company, so that the interviewees can understand the purpose of the study and the content of the interview and give more professional and valuable information. In this study, 23 interviewees were selected, and all of them satisfied the basic requirements, and the basic information of the interviewees is shown in Table 3.

**Table 3:** Basic information about the interviewees.

Basic information	Attribute	Number	Percentage
Gender	Male	17	73.91%
	Female	6	26.09%
Educational Background	Bachelor	6	26.09%
	Master	15	65.21%
	Ph.D.	2	8.70%
Position	Grassroot Management	5	21.74%
	Middle Management	10	43.48%
	Senior Management	6	26.08%
	Consultant	2	8.70%
Work Experience	3 years and below	3	13.04%
	4 to 10 years	14	60.87%
	11 to 15 years	4	17.39%
	15 years or more	2	8.70%

#### 3.2.2. Interview data collection

Before conducting the formal interviews, a more detailed semi-structured interview outline was developed, as shown in Table 4, based on this study’s research questions and objectives, in conjunction with the relevant literature and expert counsel. This study employed semi-structured interviews with 23 interviewees, each lasting at least 60 minutes, and interviews were conducted from 5 January to 29 January 2023. The interviews centered on three topics of supply chain resilience: comprehension, state, and elements. At the same time, the interview questions are flexible according to the actual situation in the interview process, and the interviewees are not bound to the interview outline. They are allowed to play freely and ask their questions. In addition to this, it is also necessary to use the STAR rule to extract information from the four aspects of the situation, task, action, and result.

**Table 4:** Interview outline.

No.	Interview topic	Interview Title
1	Comprehension of supply chain resilience	Do you comprehend the meaning of supply chain resilience?
2		What are the disturbances that supply chains encounter in their daily operations? What are the specific situations of disturbances? Why do these situations occur?
3	The state of supply chain resilience	What measures does your company take to enhance supply chain resilience? How effectively are these measures implemented?
4		What actions does the company take in response to the most recent supply chain disruption? What is the result? Is the supply chain ultimately restored? If so, how long does recovery take?
5		After experiencing supply chain disruptions, do companies learn from their mistakes and strengthen supply chain resilience? If so, please explain.
6	Elements of supply chain resilience	In your own experience, what elements do you believe contribute to supply chain resilience?
7		What role do you believe these elements will play in the three phases of supply chain resilience?
8		Which of these elements do you consider to be the key ones? Why?

## 4. Data analysis

The interview recordings are transcribed verbatim into textual materials, and the qualitative analysis software NVivo20 is utilized to guarantee a standard, regular, and systematic coding procedure. In this study, 19 of the 23 profiles are chosen randomly for formal coding, and four profiles are set aside for saturation testing as a basis for the re-validation of the identified and summarised results.

### 4.1. Open coding

Open coding is the process of conceptualizing and categorizing original data. Firstly, the interview text is conceptualized sentence by sentence without subjective bias or preconceptions, and the original meaning is retained as far as possible. Secondly, the initial concepts repeated less than three times are eliminated, and the categories are refined. Finally, 53 concepts are obtained, and 21 categories are abstracted from the concepts, as shown in Table 5.

**Table 5:** Open coding results.

Category	Original data and initial concept
Product supply range	We do not know exactly what categories of products consumers prefer, so companies produce various products and may have multiple designs for the same product. Therefore, having multiple products or solutions to satisfy your customers’ needs is more accessible. In addition, when a company produces a greater variety of products, it can reduce its reliance on a single supplier or region and a single supply chain. (Product category) Some disruptions occur because of insufficient demand; therefore, customization is essential. The customer has needs, I customize based on the customer’s needs, and I attempt to develop something for the customer that is not currently available on the market. I will have many long-term consumers in the future. (Satisfy personalized need)
Product supply time	The response efficiency of companies in the service process is one of the most critical factors that customers consider, as companies must be customer-centric. When customers’ needs are communicated in the first place, customers will think that companies pay enough attention and are often more willing to buy more products. (Customer demand response time) The delivery schedule of the orderer changes frequently, and we have so many customers. I recall the last time many customers wanted to receive the goods later. These things piled up in our warehouse, and we must find a way to deal with these stocks. There is no way to continue producing something else for a short time, so the company must be adaptable and able to deal with this issue. (Deliver resilience)
Product quantity flexibility	If suppliers cannot accurately forecast customer demand, they may run out of inventory and not provide products or services timely, impacting the supply chain. Moreover, if demand exceeds expectations, suppliers may experience stockouts or production delays, which can cause supply chain disruptions. (Adapt to changes in customer demand volume)
Product supply efficiency	The company’s operation will encounter some major customers defaulting, significantly impacting the supply chain. The first is that there is no more order quantity, which will lead to production line stoppage and affect the regular operation of upstream suppliers; the second is that it will cause an inventory backlog, which may lead to supply chain disruption if the warehouse capacity is limited. (Order missing rate) Sometimes the supply is less than the demand, the company’s production schedule is tight, and the product must be delayed because it must be transported to downstream companies, which takes time. Consequently, this backorder disruption will be progressively amplified along the supply chain’s nodes. (Order delay rate) In order to reduce the risk of delivery delays, companies may choose to receive raw materials earlier, mainly when supplies are limited. Companies dread a scenario in which the factory’s machines can operate routinely. However, there is a lack of raw materials, resulting in production lines ending, production schedules not being met, and orders not being fulfilled by the due date. (Order delivered early)
Human resource guarantee	I have observed several businesses with training as a routine operation but nothing practical. In reality, staff training is a form of adult education that is best aligned with the principle of voluntary staff and position-based training. My company can achieve this, and its employees are motivated to acquire new skills and put them into practice. They can respond flexibly to unanticipated events, such as resolving staffing shortages in emergency production. (Multi-skilled workforce) Some employees at my company are rotated to adapt to different work environments and requirements. On the one hand, they want employees to gain more insight and hope they can learn new things from different positions; on the other hand, they want to reduce the number of errors made by employees. (Employee behavior flexibility) Today, the business environment is so volatile that the company takes risk management culture seriously, and employees have contingency plans for completing essential tasks. In addition, the leadership is conservative, and employees are consciously risk-averse in the process of designing solutions. (Employee risk awareness)
Procurement guarantee	Multiple supply chain means that your supply sources are multi-channel, sourcing the same kind of materials from several different suppliers, which can spread the procurement risk to multiple suppliers and regions, thereby decreasing the likelihood of supply chain disruptions by reliance dependence on any one supplier or region. (Multiple sourcing) The company will choose one main supplier because it is less costly and has a backup plan. If the main supplier cannot provide the required materials, it will use an alternative solution to be prepared. (Backup supplier) Contracts are still vitally essential; with the contract, your supplier will be less likely to default, and even if they do, you can still recover liquidated damages. Although the liquidated damages are significantly less than your losses, they are still preferable to nothing. (Contractual recourse option) We generally rank suppliers’ reliability based on delivery punctuality and product quality and select reliable suppliers to avoid problems with upstream suppliers’ product delivery affecting normal production. (Supplier reliability)
Production equipment guarantee	If the production equipment is of good quality and is regularly maintained and serviced, then doesn’t this reduce the probability of disruptions within the supply chain caused by routine operational failures of the production equipment? (Equipment reliability) Capacity redundancy means the company has spare capacity to meet additional customer demand, reducing the risk of internal supply chain disruptions caused by machine load carrying when demand surges. (Capacity redundancy)
Logistic guarantee	Suppliers, manufacturers, and retailers all have additional inventory because we cannot guarantee that upstream companies will deliver on time, and the flow of goods can be disrupted. While inventory sometimes increases our costs, we also have additional costs when we run out of stock. (Inventory redundancy) Logistics expenses play an essential part in the company’s expenses. Part of the company’s goods are transported by ourselves, and part is outsourced to third-party logistics companies for transportation, mainly by road. There are backup options; for example, air freight is also used for urgent shipments. (Alternative transportation channels) Because there is a problem with on-time delivery, we will only search for logistics businesses with a high reputation. We cannot produce effectively if the logistics company provides poor service and cannot deliver on time. (Logistics company reputation)
Cooperation closeness	Supply chain construction is hierarchical, and we are graded according to A, B, and C. Class A is a long-term cooperation, such as the whole market supply being less than the demand. The supply is tight now, and you can prioritize supply to class A partners. (Long-term cooperation relationship) Companies have a common goal between them, to be able to share certain risks in a complex environment. If a problem occurs in one of the links, resulting in supply chain disruption, the two sides can negotiate together to solve the problem and share the loss. In the event of divergent interests, companies sharing benefits will be more willing to cooperate and support and help each other, which can promote the stability of the supply chain. (Shared risk and shared benefits) We also often discuss with upstream and downstream partners together because the upstream does not give goods, we have no way to produce, the downstream is not clear about our situation, they also have no way to produce in the time of shortage of goods, the supply chain upstream and downstream is best to make decisions together to avoid production awkward this situation. (Joint decision-making)
Information sharing degree	When the supply chain is disrupted, for example, the supply is less than the demand, and you do not know the actual inventory of upstream suppliers, do you dare to make your next plan at will? You dare not because the risk is too significant. However, if you know the actual situation of upstream suppliers, you can boldly proceed. (Sharing information) We will receive and process much information daily because of frequent contact and mutual trust. Long-term partners can reduce duplication of work and unnecessary communication and improve each other’s work efficiency. (Mutual trust)
Collaboration partner	Different types of collaboration partners may take different amounts of time to align. For example, changing raw material suppliers may take longer to achieve because factors such as quality, price, and delivery time of raw materials need to be considered. However, changing logistics service providers may take less time because logistics services can be transferred quickly. (Time to change collaboration partner) In order to find a suitable new collaboration partner, market research, evaluation, and screening may be required, and these processes will be labor-intensive and costly. Moreover, once found, negotiations, contracts, and agreements with the new collaboration partner will be required, and these processes will require the payment of related costs. (Cost to change collaboration partner) Different collaboration partners can provide different products, services, and technologies, thus reducing the risk of supply chains relying on a single supplier or product. When some collaboration partners in the supply chain are adversely affected, other collaboration partners can provide alternative or substitute solutions, thus reducing the loss of supply chain disruption. (Collaboration partner number)
Information technology level	Now we all work together by computer. Our company uses an intelligent logistics platform to automate and collaboratively manage the logistics process by monitoring and tracking data from logistics and production in real time. This allows us to improve logistics efficiency and accuracy while identifying supply chain problems and making rapid responses and adjustments. (IT infrastructure) Big data technology is a must-have for businesses. It can be used to analyze data such as order volume, transit time, and inventory levels, thus helping companies to predict future demand and risk. For example, with this technology, I can find some products with long delivery lead times, which may be an undetected problem, so I have to find ways to shorten the delivery lead time and avoid backorders. (Big data technology)
Information response capability	When Samsung launched one of its cell phones, there was a safety issue when the battery exploded. However, Samsung’s supply chain was too slow to respond, and despite knowing the problem existed, it decided to bring the product to market, which led to a massive product recall and loss. This issue seriously affected Samsung’s brand value and market share. (Information response speed) When a company has a wide range of information responses, it responds to the information of its horizontal partners at the same level and the subjects of each link upstream and downstream vertically. It can quickly understand the scope and severity of the problem and thus take appropriate measures to restore the stability and resilience of its supply chain.(Information response range)
Information distribution accuracy	Accurate information can improve the transparency and integrity of companies. We are familiar with the bullwhip effect, and it is because of accurate information that we do not know the actual situation, resulting in information distortion by magnification. Moreover, if there is a long-term misinformation situation, companies do not trust each other, let alone talk about cooperation. (Supply chain visibility) There are differences between the information you receive and the information you understand, as well as between the information you intend to deliver and the information you actually deliver; therefore, the company should adopt scientific methods and tools to analyze and deliver information to avoid personal subjective factors and biases in delivering wrong information and make wrong decisions. (Information distribution mechanism) Because management has to make decisions based on information, if management receives inaccurate information about market trends, customer needs, and competitors, then management will make wrong decisions, resulting in business losses or missed business opportunities, so management attaches great importance to the accuracy of information. (Management attention)
Financing capability	Generally speaking, if the enterprise is in an essential part of the supply chain or the supply chain core enterprise is your investor or a strategic partner, it will be willing to guarantee your loan because you still have value for it to use. (Supply chain finance) Entities are better at financing because you have the equipment, plants, and other physical objects that can be collateralized, the total quantity of assets will generally be higher, and the asset-light operation of the company’s loans may be relatively tricky. (Total assets) The equity cannot be complicated, and we should secure 2/3 or 50% or more. When you require financing, others will discuss the fixed with you. If the equity is dispersed, the financier feels useless to talk to you because you still have to hold a shareholders’ meeting. (Equity structure) Whether you seek a bank loan or private financing, you will assess the company’s credit level, such as the loan record and default record. Otherwise, who dares to borrow money? (Credibility)
Profit capability	The greater a company’s gross margin, the greater its production and sales profits and profitability. For instance, a high-end electronics manufacturer like Apple may have a gross profit margin of 40% or more. A manufacturer of low-priced products may have a gross profit margin of only 10%. I have a high gross profit margin to improve supply chain resilience while making money. (Gross margin) We sell a variety of products, with some selling particularly well. The monthly increase in sales allows me to earn more money. Therefore, I can invest additional resources to prevent supply chain disruptions. (Sales revenue growth rate) A company’s excessive inventory turnover days are incredibly detrimental. For example, it solidifies the company’s capital; excessive inventory will cause a significant amount of capital to be frozen in inventory, and working capital is getting short. Excessive inventory lengthens the storage period and increases the likelihood of inventory loss. (Inventory turnover day)
Price resilience	We are all aware that price reductions can increase demand. Tesla, for instance, has several unique features in the management and design of the car, and the cost of producing the car is low, something that many car companies cannot do; as a result, they can reduce the price and stimulate demand. (Cost control) Doing retail supply chain can not avoid product homogenization; you sell a product that others also sell, and you must have a price advantage to attract customers and obtain a competitive advantage. (Price advantage)
Market position	Your market share is high, which indicates that consumers embrace your products to a high degree and are compelled to purchase them. At the same time, you have strong bargaining power in the supply chain, so your ability to generate profits is also substantial. (Market share) Customers now have higher expectations for product quality, and in general, the quality and credibility of a well-known brand are not poor. Therefore, if your product is well-known and others have confidence in you, it will sell well. (Brand awareness)
Learning organization	After supply chain disruptions, executives must meet for reflection, and some departments are criticized for poor performance. Each department must essentially write a summary, considering what can be done to enhance the business operation process and compensate for shortcomings. (Learning from experience) Due to the rapid development of the times, specific knowledge is no longer sufficient; therefore, lifelong learning is now advocated, and members of our company will assist each other and share their knowledge and experience so that we can continue to advance. (Knowledge sharing culture) Sometimes we also have joint training with other businesses, on the one hand, because some training experts charge too much, so we can reduce costs; on the other hand, we can communicate and learn from one another, share our experiences, and contact each other. (Inter-organizational experience sharing)
Collaboration innovation	Product innovation is highly challenging. Although we have our R&D team and invest in R&D, we must focus on one part. We should find partners, integrate their technology, conduct secondary development, and design new products to meet emerging market demands. (Collaboration technology innovation) By collaborative innovation, we do not only refer to technical innovation, as is commonly misconstrued. In reality, it entails extremely complex factors, such as innovation in marketing, transportation, and other operational processes, which are crucial for enhancing consumer satisfaction and should be discussed with partners. (Collaboration management innovation)
Product development	As we all know, the product life cycle is shortening nowadays. A product that explodes may go cold after a time, which requires companies to continue to invest in developing new products, although it costs a lot; otherwise, how can they keep making money? (Develop new product cost) Some raw materials may not be delivered on time. Then you must find a way to develop an alternative product that bypasses this raw material in the shortest possible time to maintain market share and minimize the economic and reputational losses caused by late delivery. In order to take advantage of the business opportunity, you must create a new product as soon as possible; otherwise, it will be too late. (Develop new product time)

### 4.2. Axial coding

The objective of axial coding is to analyze the logical relationships between various categories, to further summarise the categories derived from open coding, and to develop the main categories. Based on open coding, the logical relationships and evolutionary patterns between the 21 categories are compared. Six main categories are identified, namely product supply resilience, resource resilience, partner resilience, information response resilience, capital resilience, and knowledge resilience, as shown in Table 6.

**Table 6:** Axial coding results.

Main Category	Category
Product supply resilience	Product supply range, Product supply time, Product quantity flexibility, Product supply efficiency
Resource resilience	Human resource guarantee, Procurement guarantee, Production equipment guarantee, Logistic guarantee
Partner resilience	Cooperation closeness, Information sharing degree, Collaboration partner
Information response resilience	Information technology level, Information response capability, Information distribution accuracy
Capital resilience	Financing capability, Profit capability, Price resilience, Market position
Knowledge resilience	Learning organization, Collaboration innovation, Product development

Product supply resilience reflects the characteristics of supply chain output results and delivery, reflecting the company’s capability to respond to changes in market demand and provide services to customers and meet their needs, which is directly related to customer satisfaction. Resource resilience indicates that the company has a certain level of flexibility and redundancy in its human resources, procurement, production equipment, and logistics, which can absorb environmental disturbances and reflect its capability to deal with internal and external environmental uncertainties. Partner resilience implies that the company can change and modify its partners and operate collaboratively in response to environmental changes and demands to better adapt to the environment. Information response resilience reflects the capability of supply chain members to receive, process, and provide feedback on information to provide information support for timely action, allowing the company to respond quickly to changes in the external environment. Capital resilience reflects the economic strength of supply chain members to provide capital for supply chain recovery and growth from disruptions. Knowledge resilience is the capability of the company to learn from disruptions to improve operational efficiency while updating technology and developing new products to satisfy customer demand more efficiently and effectively, thereby enabling the supply chain to recover and grow.

### 4.3. Selective coding

Selective coding is the process of identifying core categories through an exhaustive analysis of all categories, analyzing the relationship paths between core categories and other categories, and developing a theoretical model. This study identified the core category of "key elements of supply chain resilience" through an in-depth analysis of 53 concepts, 21 categories, and six main categories, and the typical relationship structure is depicted in Table 7.

**Table 7:** Typical relationship structure.

Typical Relationship Structure	The connotation of relationship structure	Original data support
Product supply resilience ↓ Supply chain resilience	Product supply resilience enables the supply chain to respond flexibly to changes in customer demand, thereby enhancing supply chain resilience.	Different customers may not ask for the same things and are beginning to pursue personalization. How to achieve personalization at a low cost is a problem that companies need to solve in conjunction with their situation.
Resource resilience ↓ Supply chain resilience	Resource resilience provides security for supply chain operations and reduces the probability of disruptions, thereby enhancing supply chain resilience.	Sometimes suppliers have tight schedules and can’t deliver on time, but I have backup suppliers to avoid this disruption.
Partner resilience ↓ Supply chain resilience	Partner resilience enhances supply chain resilience by keeping the supply chain operational during disruptions and reducing losses.	Partners generally trust each other and have a joint interest base, so they will work with each other to avoid supply chain disruption or reduce supply chain disruption losses.
Information response resilience ↓ Supply chain resilience	Information response resilience improves supply chain resilience by enabling supply chain members to access and transfer information effectively and respond quickly to changes in the external environment.	We are now emphasizing the information gap because we must make decisions based on the information we already have. Making decisions based on wrong information may lead to product delivery delays, cost increases, missed market opportunities, and other problems, leading to supply chain disruptions.
Capital resilience ↓ Supply chain resilience	Capital resilience provides capital support for supply chains to recover and grow from disruptions, thereby increasing supply chain resilience.	Because during the interruption, the company can not deliver normally, resulting in downstream payment can not be recovered. If the company wants to resume normal operations, it will have to spend more, and this is when capital is significant because you do not have enough funds.
Knowledge resilience ↓ Supply chain resilience	Knowledge resilience enables supply chains to learn from disruptions and optimize operational processes and supply chain structures through collaborative innovation, thereby increasing supply chain resilience.	Communication and reflection between the upstream and downstream of the supply chain can enable supply chain members to better understand market demand and technology trends and work together to develop new products to meet customer needs.

**Fig 2:**
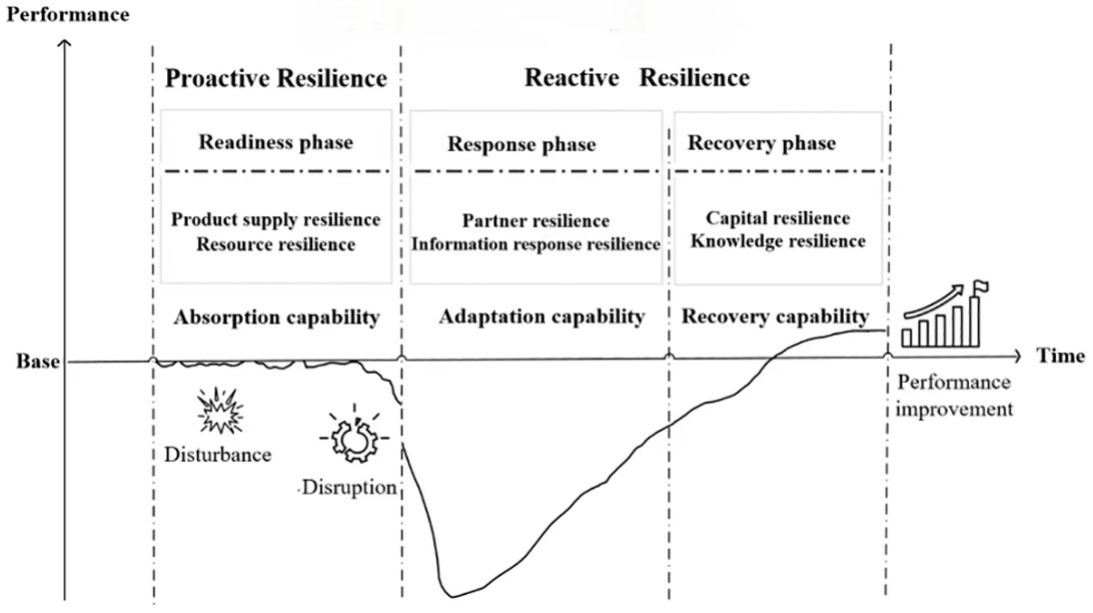
Key elements and formation mechanisms of supply chain resilience.

As shown in Fig 2, combined with the previous concepts, characteristics of supply chain resilience, and the results of coding, the formation mechanism of supply chain resilience can be explained as follows: the formation of supply chain resilience is a reflection of the integration of the capability of supply chain resilience (absorption, adaptation and recovery capability), the characteristics of dynamic evolution, ambidexterity (active resilience and reactive resilience) and phases (readiness, response and recovery phases). Before the supply chain disruption, that is, the readiness phase of supply chain resilience, the company primarily improves the absorption capability of supply chain resilience through product supply resilience and resource resilience, such as backup suppliers, inventory redundancy, multiple transportation channels, and other measures to absorb external disturbances, which exemplifies proactive resilience. As external disturbances intensify and the absorption capability fails, the supply chain is disrupted and enters the response phase. The company primarily increases the adaptability of supply chain resilience through partner resilience and information response resilience, which makes the supply chain respond quickly to the disruption and minimize the loss of disruption. Upon completion of the response phase, the supply chain gradually enters the recovery phase. With the capital support, the company collaborates with supply chain members to complete the recovery plan, improve the recovery capability of supply chain resilience, and progressively restore the supply chain. Concurrently, reflect on the causes of disruption, learn from disruption, and achieve growth. The capability to adapt and recover is a reflection of reactive resilience. In conclusion, product supply resilience, resource resilience, partner resilience, information response resilience, capital resilience, and knowledge resilience are the key elements that contribute to the formation of supply chain resilience, and they have different effects on different capabilities and different phases of supply chain resilience.

### 4.4. Theoretical saturation test

The theoretical saturation test is a coding analysis of the set-aside data to test for the emergence of new concepts and categories in the data. If new concepts and categories emerge, the data collection must be expanded for coding until no new concepts and categories emerge. This study randomly selects 19 of the 23 profiles for formal coding, while the remaining four are reserved for saturation testing. The absence of new concepts and categories after the test demonstrates that the theory developed in this paper is essentially close to saturation and that no further sample expansion is required.

## 5. Model verification

### 5.1. Research hypothesis

According to the grounded theory analysis, the key elements of supply chain resilience are product supply resilience, resource resilience, partner resilience, information response resilience, capital resilience, and knowledge resilience. The theoretical model is further validated and analyzed using a structural equation model to assess its rationality and reliability. The structural equation model is constructed with product supply resilience, resource resilience, partner resilience, information response resilience, capital resilience, and knowledge resilience as exogenous latent variables and supply chain resilience as endogenous latent variables. The following hypotheses are put forward:

H1: Product supply resilience positively influences supply chain resilience;
H2: Resource resilience positively influences supply chain resilience;
H3: Partner resilience positively influencing supply chain resilience;
H4: Information response resilience positively influences supply chain resilience;
H5: Capital resilience positively influences supply chain resilience;
H6: Knowledge resilience positively affects supply chain resilience.

### 5.2. Questionnaire design

Regarding the design of the measurement items, each exogenous latent variable mainly referred to the results of the previous spindle coding, as shown in Table 8. Regarding reliability and validity measures, the Likert 7-point scale is superior to the Likert 5-point scale [59, 60]. Therefore, the Likert 7 subscale is used with a scale of 1–7, ranging from strongly disagree to strongly agree.

**Table 8:** Questionnaire scales.

Variable	Item	Measurement Question	Source
Product supply resilience (PSR)	PSR1	We offer a wide range of products to meet customers’ needs regarding product variety.	Axial coding results
	PSR2	We can meet the personalized needs of customers.	
	PSR3	We can respond to changes in customer demand timely.	
	PSR4	We can adjust the delivery time according to the customer’s requirements.	
	PSR5	We can adjust our production volume according to changes in customer demand.	
	PSR6	Our products are supplied effectively and delivered on time or early.	
Resource resilience (RER)	RER1	Our employees are risk-aware and perceptive.	Axial coding results
	RER2	Our employees can adapt to different work environments and work requirements.	
	RER3	Our employees have specialized training and are multi-skilled.	
	RER4	We use multiple channels for sourcing.	
	RER5	We have multiple backup suppliers.	
	RER6	We evaluate the reliability of suppliers and choose the best ones.	
	RER7	Our production equipment is reliable and can guarantee normal production.	
	PER8	We have a reliable logistics system to guarantee transportation activities.	
Partner resilience (PAR)	PAR1	We have a high degree of cooperation with our supply chain members.	Axial coding results
	PAR2	We have a high level of information sharing with our supply chain members.	
	PAR3	We have a high level of trust with our supply chain members.	
	PAR4	We can adjust our collaboration partners timely.	
Information response resilience (IRR)	IRR1	We have advanced information technology.	Axial coding results
	IRR2	We can respond to information quickly.	
	IRR3	We can respond to information from different sources.	
	IRR4	We have a high level of accuracy in information distribution.	
Capital resilience (CAR)	CAR1	We have a strong financing capability.	Axial coding results
	CAR2	We have a strong profit capability.	
	CAR3	Our products have strong price advantages.	
	CAR4	We have a high market share.	
	CAR5	We have high brand awareness.	
Knowledge resilience (KNR)	KNR1	We strive to build learning organizations.	Axial coding results
	KNR2	Our new technologies can be developed and utilized.	
	KNR3	We master newer technologies with a distinct technical paradigm than traditional technologies.	
	KNR4	We actively work with supply chain members to develop new technologies	
	KNR5	We actively work with supply chain members to improve and innovate operational processes.	
	KBR6	We actively develop new products to satisfy market demands.	

### 5.3. Questionnaire pre-survey

Since the design of the scale is based on axial coding, the scale needs to be validated. The minimum sample size for the pretest should equal three times the number of measurement items on the scale [61]. The number of measurement items in this study’s pretest questionnaire is 33, so the minimum sample size for the pretest should be 99. The questionnaires were collected from 6 March to 27 March 2023, and the pre-survey of this study yields 143 questionnaires, of which 109 are valid, for an efficacy rate of 76.22%; the valid questionnaires collected satisfy the minimum sample size requirement of 99 for the pretest. This study uses SPSS 26 statistical analysis software for data processing and exploratory factor analysis.

#### 5.3.1. Reliability test

In this research, Cronbach’s α coefficient is used to measure reliability. Reliability is an indicator used to evaluate the consistency and stability of measurement results. In general, Cronbach’s α values between 0.7 and 0.8 have high reliability, and values greater than 0.8 are considered to have excellent reliability. As shown in Table 9, the overall reliability of the questionnaire is 0.930, and the reliability of each dimension ranged from 0.845 to 0.892, which is greater than the 0.7 standards for judging reliability [62]. The test results indicate that the scale’s reliability is high, and the variables have high internal consistency.

**Table 9:** Pre-survey reliability analysis.

Variable	Item	CITC	CAID	Cronbach’s α
Product supply resilience	PSR1	0.633	0.885	0.891
	PSR2	0.712	0.872	
	PSR3	0.742	0.867	
	PSR4	0.787	0.860	
	PSR5	0.651	0.881	
	PSR6	0.742	0.867	
	RER1	0.681	0.877	0.892
	RER2	0.325	0.915	
	RER3	0.754	0.870	
	RER4	0.747	0.871	
	RER5	0.821	0.863	
	RER6	0.783	0.868	
	RER7	0.734	0.874	
	PER8	0.601	0.885	
Partner resilience	PAR1	0.732	0.821	0.863
	PAR2	0.675	0.840	
	PAR3	0.742	0.814	
	PAR4	0.710	0.827	
Information response resilience	IRR1	0.773	0.803	0.865
	IRR2	0.702	0.832	
	IRR3	0.595	0.874	
	IRR4	0.791	0.795	
Capital resilience	CAR1	0.726	0.866	0.889
	CAR2	0.719	0.868	
	CAR3	0.728	0.865	
	CAR4	0.763	0.857	
	CAR5	0.715	0.868	
Knowledge resilience	KNR1	0.689	0.808	0.845
	KNR2	0.275	0.883	
	KNR3	0.661	0.813	
	KNR4	0.769	0.791	
	KNR5	0.695	0.806	
	KBR6	0.699	0.805	
General cronbach’s α			0.928	

#### 5.3.2. Initial scale purification

In this study, the quality of the items is measured by the Corrected-Item Total Correlation (CITC) and Cronbach’s α coefficient after the item is deleted (CAID), and the combination of the two could be used to determine the appropriateness of an item. The CITC value can be used to characterize the overall correlation between a item and its dimension, and the threshold value for the CITC coefficient is typically 0.4. The items with CITC below 0.4 and CAID are more excellent than Cronbach’s α coefficients of the dimension to which they belonged [63–65]. Table 9 displays the results of the scale’s CAID and CITC. According to the calculated results, the CITC values of item RER2 of the resource resilience dimension and item KNR2 of the knowledge resilience dimension are less than 0.4, and Cronbach’s α coefficient of the respective dimension would increase after the deletion of the item. Consequently, the elimination of RER2 and KNR2 is considered.

#### 5.3.3. Exploratory factor analysis

Before conducting exploratory factor analysis, it is necessary to conduct the Kaiser-Meyer-Olkin (KMO) test and Bartlett’s sphericity test to determine whether the bias correlation between variables is sufficiently significant and whether the correlation coefficient matrix is a unit matrix, respectively. The closer the KMO value is to 1, the stronger the correlation between variables, and it is generally accepted that if the KMO statistic is more significant than 0.70 and Bartlett’s sphericity test reaches a significance level of 0.05 or higher, the model is more appropriate for factor analysis. As shown in Table 10, the KMO measure for this study is 0.844, and the P-value of Barth’s spherical test is less than 0.001, indicating some structure and correlation between the original variables. Combining these two factors, the data from this investigation are therefore suitable for factor analysis.

**Table 10:** KMO and Bartlett’s Test.

**Kaiser-Meyer-Olkin Measure of Sampling Adequacy.**		**0.832**
Bartlett’s Test of Sphericity	Approx. Chi-Square	2335.186
	df	465
	Sig.	0.000

This study uses principal component factor analysis, the remaining 31 items are orthogonally rotated, and six factors with eigenvalues greater than one are extracted, yielding a cumulative interpretation variance of 75.241%, which is a high degree of explanation, and the analysis results are shown in Table 11. The first factor loadings are RER1~ RER8 items (RER2 has been removed), which have higher loading coefficients on the resource resilience factor; the second-factor loadings are PSR1~ PSR6 items, which have higher loading coefficients on the product supply resilience factor; The third-factor loadings are items KNR1~ KNR6 (KNR2 has been eliminated). These five items have high loading coefficients on the knowledge resilience factor; the fourth-factor loadings are CAR1~ CAR5 items, which have high loading coefficients on the capital resilience factor; the fifth-factor loadings are IRR1~ IRR4 items, which have high loading coefficients on the information response resilience factor; and the sixth-factor loadings are PAR1~ PAR4 items, and these four indicators have high loading coefficients on the knowledge resilience factor.

**Table 11:** Pre-survey exploratory factor analysis results.

Item	Component					
	1	2	3	4	5	6
PSR1	0.140	**0.647**	0.200	0.243	0.057	0.024
PSR2	0.113	**0.707**	0.256	0.173	0.200	0.089
PSR3	0.067	**0.767**	0.287	0.177	0.043	0.062
PSR4	0.157	**0.874**	0.000	0.025	0.099	0.042
PSR5	0.164	**0.755**	0.069	-0.090	0.131	0.125
PSR6	0.065	**0.832**	-0.022	0.161	0.053	0.136
RER1	**0.714**	0.052	0.136	0.176	0.064	0.187
RER3	**0.805**	0.022	0.150	0.079	0.143	0.083
RER4	**0.781**	0.185	0.224	0.185	0.006	0.027
RER5	**0.865**	0.171	0.036	0.130	0.102	0.030
RER6	**0.801**	0.076	-0.005	0.250	0.098	0.173
RER7	**0.826**	0.135	0.049	0.020	0.118	0.014
PER8	**0.679**	0.122	-0.211	0.104	0.036	0.190
PAR1	0.030	0.060	0.036	0.065	0.009	**0.875**
PAR2	0.155	0.175	0.161	0.179	0.338	**0.692**
PAR3	0.165	0.202	0.080	-0.005	0.067	**0.825**
PAR4	0.251	0.017	0.189	0.024	0.040	**0.800**
IRR1	0.120	0.024	0.231	0.178	**0.846**	0.023
IRR2	0.077	0.183	0.021	0.203	**0.811**	0.086
IRR3	0.352	0.162	0.161	0.060	**0.611**	0.218
IRR4	0.071	0.165	0.194	0.188	**0.837**	0.069
CAR1	0.183	0.098	0.272	**0.728**	0.201	0.005
CAR2	0.067	0.230	0.074	**0.777**	0.160	0.078
CAR3	0.137	0.126	0.147	**0.792**	0.094	0.094
CAR4	0.192	0.108	0.222	**0.806**	0.080	-0.002
CAR5	0.272	0.026	0.136	**0.742**	0.165	0.070
KNR1	0.060	0.107	**0.779**	0.218	0.038	0.129
KNR3	-0.001	0.242	**0.698**	0.208	0.094	0.061
KNR4	0.057	0.110	**0.780**	0.204	0.212	0.220
KNR5	0.160	0.041	**0.807**	0.178	0.072	0.130
KNR6	0.043	0.150	**0.794**	0.016	0.203	-0.036
The cumulative interpretation variance	31.426%	42.42%	51.184%	58.848%	65.024%	70.724%

### 5.4. Formal questionnaire

#### 5.4.1. Questionnaire design, distribution, and collection

The scale of six key elements has been explored previously, and the supply chain resilience (SCR) variable is presented to validate the influence of six key elements on it. Supply chain resilience is measured concerning the scale of Ambulkar et al. [66] as We can cope with changes brought by the supply chain disruption (SCR1); We can adapt to the supply chain disruption easily (SCR2); We can provide a quick response to the supply chain disruption (SCR3); We can maintain high situational awareness at all times (SCR4). The rest of the scale design is the same as the pre-survey.

From 13 April to 15 May 2023, 543 questionnaires were collected, 409 of which are valid, for a valid recall rate of 75.32 percent. For credible parameter estimates, the ratio of a sample size to scale items must be at least 5:1, and a ratio of 10:1 or close to 10:1 is generally required to assure the validity of the significance test [67]. There are 35 items on the scale, and the ratio of valid questionnaires to items is 11.69:1, greater than the minimum of 10:1.

#### 5.4.2. Reliability test

Cronbach’s α coefficient can be used to test reliability. As shown in Table 12, the study results indicated that Cronbach’s α coefficient values for each scale dimension were above 0.7, higher than the 0.7 standards for judging reliability [62].

**Table 12:** Cronbach’s α coefficient.

Variable	Item number	Cronbach’s α coefficient	General
Product supply resilience	6	0.891	0.943
Resource resilience	7	0.904	
Partner resilience	4	0.845	
Information response resilience	4	0.879	
Capital resilience	5	0.838	
Knowledge resilience	5	0.849	
Product supply resilience	4	0.821	

#### 5.4.3. Exploratory factor analysis

The KMO measure of the formal survey is 0.942, and the P-value of Barth’s spherical test is less than 0.001; the data from this investigation are therefore suitable for factor analysis. Using principal component factor analysis, orthogonal rotation is performed on 35 items, and seven factors are extracted with a cumulative interpretation variance of 65.987%, indicating a high degree of explanation; the analysis results are presented in Table 13.

**Table 13:** Formal survey exploratory factor analysis.

Item	Component						
	1	2	3	4	5	6	7
PSR1	0.162	**0.775**	0.144	0.147	0.116	0.097	0.168
PSR2	0.132	**0.761**	0.065	0.090	0.082	0.160	0.040
PSR3	0.094	**0.755**	0.102	0.141	0.095	0.087	0.159
PSR4	0.133	**0.771**	0.105	0.133	0.047	0.143	0.115
PSR5	0.179	**0.752**	0.048	0.126	0.111	0.027	0.017
PSR6	0.121	**0.710**	0.086	0.117	0.160	0.161	0.147
RER1	**0.741**	0.137	0.096	0.185	0.254	0.167	0.118
RER3	**0.718**	0.185	0.067	0.189	0.135	0.140	0.088
RER4	**0.711**	0.115	0.176	0.164	0.175	0.151	0.136
RER5	**0.748**	0.086	0.155	0.101	0.012	0.118	0.193
RER6	**0.736**	0.108	0.114	0.168	0.071	0.193	0.098
RER7	**0.739**	0.121	0.133	0.091	0.147	0.056	0.062
PER8	**0.689**	0.220	0.091	0.161	0.126	0.122	0.144
PAR1	0.234	0.145	0.101	0.211	0.120	**0.717**	0.159
PAR2	0.244	0.224	0.151	0.134	0.099	**0.698**	0.132
PAR3	0.178	0.155	0.167	0.235	0.092	**0.712**	0.178
PAR4	0.158	0.148	0.202	0.135	0.144	**0.758**	0.101
IRR1	0.181	0.098	0.122	0.163	**0.792**	0.127	0.148
IRR2	0.168	0.151	0.073	0.060	**0.818**	0.045	0.137
IRR3	0.158	0.178	0.150	0.098	**0.794**	0.120	0.129
IRR4	0.166	0.111	0.161	0.112	**0.768**	0.127	0.115
CAR1	0.200	0.157	0.109	**0.686**	0.126	0.122	0.267
CAR2	0.142	0.128	0.104	**0.700**	0.099	0.169	0.225
CAR3	0.167	0.174	0.109	**0.688**	0.123	0.137	0.255
CAR4	0.224	0.121	0.077	**0.752**	0.062	0.122	0.088
CAR5	0.197	0.215	0.047	**0.686**	0.097	0.170	-0.168
KNR1	0.135	0.121	**0.781**	0.070	0.052	0.115	0.054
KNR3	0.105	0.182	**0.769**	0.028	0.065	0.072	0.102
KNR4	0.110	0.018	**0.760**	0.098	0.138	0.074	0.112
KNR5	0.088	0.076	**0.747**	0.140	0.084	0.102	0.135
KBR6	0.200	0.077	**0.699**	0.042	0.154	0.181	0.020
SCR1	0.330	0.266	0.182	0.168	0.214	0.175	**0.576**
SCR2	0.215	0.151	0.181	0.240	0.175	0.173	**0.669**
SCR3	0.254	0.233	0.133	0.191	0.220	0.147	**0.630**
SCR4	0.207	0.212	0.146	0.156	0.218	0.294	**0.579**
The cumulative interpretation variance	35.042%	42.137%	48.628%	54.214%	59.286%	63.125%	65.987%

#### 5.4.4. Validity test

The validity test includes content validity and construction validity, and construction validity can be divided into convergent validity and discriminant validity. In this paper, the six key elements of supply chain resilience are coded from the results of axial coding and validated by exploratory factor analysis, and the items of supply chain resilience are taken from the mature scales of existing literature. The items are pretested and validated by experts to ensure the quality of the scale content and therefore have high content validity.

Convergent validity can be examined utilizing item loading, composite reliability (CR), and average variance extracted (AVE). When the item loading is more significant than 0.6, and the CR and AVE values for each variable are more significant than 0.7 and 0.5, respectively, the scale’s convergent validity is high [68, 69]. As shown in Table 14, the minimum item loading is 0.611, which is greater than 0.6; the minimum CR value of each variable is 0.821, which is greater than 0.7; and the minimum AVE value is 0.518, which is greater than 0.5, indicating that the scale have good convergent validity.

**Table 14:** Item loadings and convergent validity tests.

Variable	Item	Unstd.	S.E.	t-value	P	Std.	CR	AVE
PSR	PSR1	1				0.835	0.892	0.579
	PSR2	0.827	0.05	16.654	***	0.741		
	PSR3	0.844	0.049	17.269	***	0.761		
	PSR4	0.85	0.048	17.629	***	0.772		
	PSR5	0.841	0.053	15.869	***	0.714		
	PSR6	0.863	0.052	16.521	***	0.736		
RER	RER1	1				0.825	0.904	0.574
	RER2	0.883	0.051	17.312	***	0.761		
	RER3	0.871	0.049	17.776	***	0.775		
	RER4	0.793	0.048	16.494	***	0.734		
	RER5	0.901	0.052	17.236	***	0.758		
	RER6	0.787	0.05	15.735	***	0.708		
	RER7	0.866	0.052	16.607	***	0.738		
PAR	PAR1	1				0.768	0.846	0.579
	PAR2	0.925	0.062	14.871	***	0.751		
	PAR3	0.932	0.06	15.421	***	0.778		
	PAR4	0.918	0.062	14.764	***	0.746		
IRR	IRR1	1				0.821	0.880	0.647
	IRR2	0.917	0.051	18.044	***	0.810		
	IRR3	0.896	0.049	18.372	***	0.822		
	IRR4	0.885	0.053	16.72	***	0.763		
CAR	CAR1	1				0.761	0.842	0.518
	CAR2	0.906	0.063	14.306	***	0.731		
	CAR3	0.874	0.059	14.725	***	0.752		
	CAR4	0.9	0.063	14.354	***	0.733		
	CAR5	0.87	0.073	11.855	***	0.611		
KNR	KNR1	1				0.762	0.850	0.532
	KNR2	0.916	0.064	14.312	***	0.738		
	KNR3	0.926	0.066	14.027	***	0.724		
	KNR4	0.914	0.065	14.062	***	0.726		
	KNR5	0.936	0.069	13.472	***	0.696		
SCR	SCR1	1				0.767	0.821	0.534
	SCR2	0.935	0.065	14.398	***	0.722		
	SCR3	0.944	0.066	14.364	***	0.720		
	SCR4	0.879	0.062	14.182	***	0.712		

The Discriminant validity criterion is that the square root of the AVE value of each variable (the main diagonal part) must be greater than the correlation coefficient between the variable and other variables [68]. As shown in Table 15, the square root of the AVE value of each variable is greater than the correlation coefficient between that variable and the other variables. Consequently, there is a high degree of discriminant validity between the items.

**Table 15:** Discriminant validity test.

Variance	AVE	SCR	KNR	CAR	IRR	PAR	RER	PSR
SCR	0.534	**0.731**						
KNR	0.532	0.529	**0.729**					
CAR	0.518	0.694	0.377	**0.720**				
IRR	0.647	0.636	0.398	0.442	**0.804**			
PAR	0.579	0.709	0.489	0.620	0.459	**0.761**		
RER	0.574	0.700	0.437	0.595	0.510	0.604	**0.758**	
PSR	0.579	0.621	0.364	0.514	0.421	0.519	0.480	**0.761**

Diagonal value: Squared root of AVE, Non-diagonal value: Correlation.

Product supply resilience (PSR), Resource resilience (PER), Partner resilience (PAR), Information response resilience(IRR), Capital resilience (CAR), Knowledge resilience (KNR), Supply chain resilience (SCR)

#### 5.4.5. Model fit and hypothesis testing

**Fig 3:**
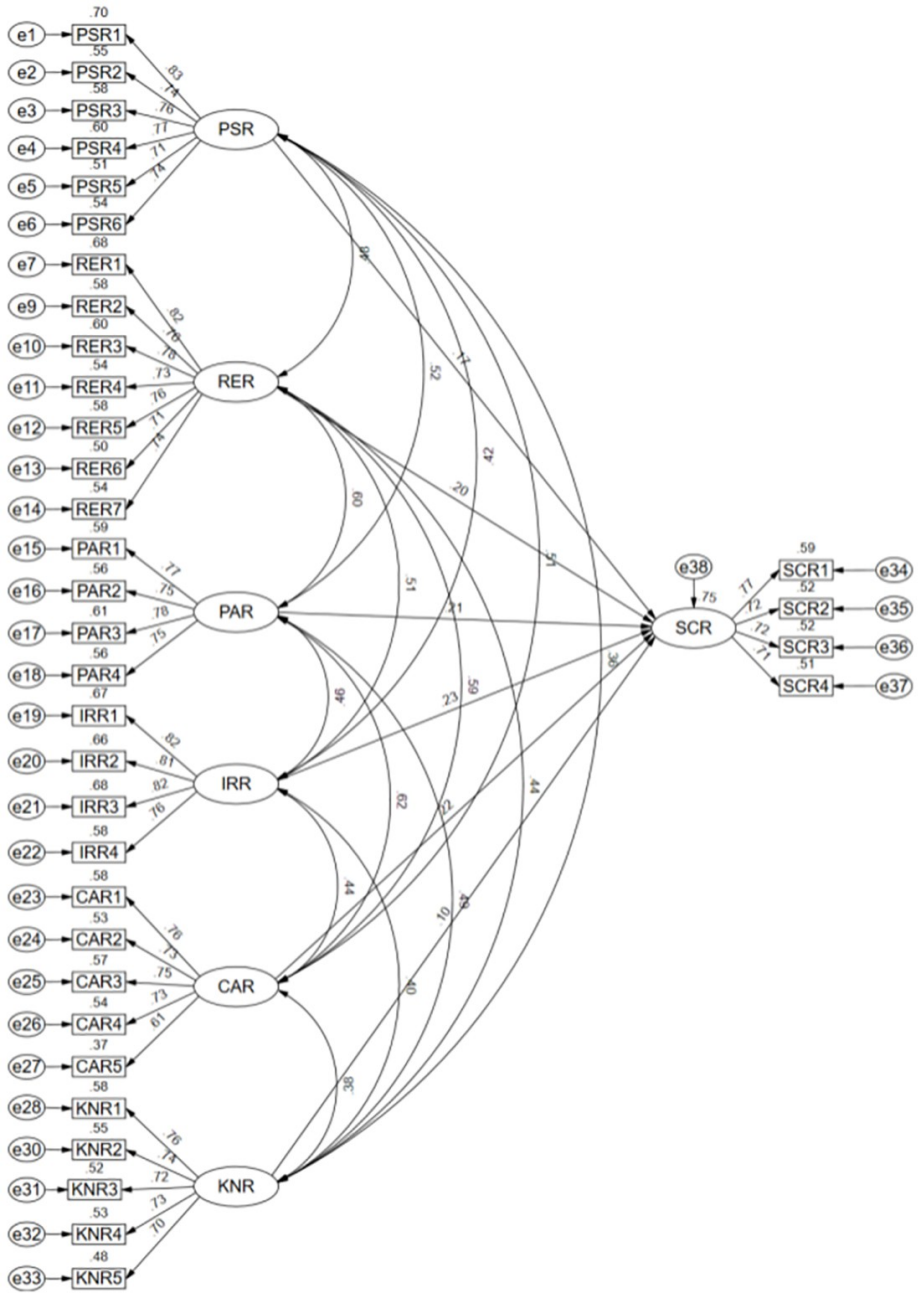
Standardized path coefficient diagram.

In this research, the Amos 28 software is used for model fitting and hypothesis testing, and Table 16 displays the fitting results of the theoretical model. Referring to the criteria of authoritative scholars, Hayduk [70], Bagozzi & Yi [71], Hu & Bentler [72], and Wu [73], all the fit indicators of this model satisfy the reference criteria, indicating that the overall fit between the data and the model is good. The results of the model’s hypothesis testing are shown in Table 17 and Fig 3. Product supply resilience, resource resilience, partner resilience, information response resilience, and capital resilience significantly positively affect supply chain resilience with path coefficients of 0.170, 0.200, 0.209, 0.229, and 0.218, respectively, and knowledge resilience positively affects supply chain resilience with a path coefficient of 0.103. In conclusion, all the six research hypotheses proposed in this paper are tested.

**Table 16:** Model fitting results.

Indicators	Value	Criteria	Conclusion	Criteria Source
CMIN	691.974	The smaller, the better		
DF	539	The larger, the better		
CMIN/DF	1.284	< 3 is perfect; < 5 is acceptable	Perfect	Hayduk, 1987
GFI	0.916	> 0.8 is acceptable; > 0.9 is good	Good fit	Bagozzi and Yi, 1988
AGFI	0.902	> 0.8 is acceptable; > 0.9 is good	Good fit	Hu and Bentler, 1998
CFI	0.979	> 0.9	Good fit	Bagozzi and Yi, 1988
TLI	0.977	> 0.9	Good fit	Wu, 2010
RMSEA	0.026	< 0.08 is perfect; < 0.1 is acceptable	Perfect	Bagozzi and Yi, 1988
SRMR	0.033	< 0.08	Good fit	Hu and Bentler, 1998

**Table 17:** Hypothesis testing results.

Hypothesis	Path			Standardized path coefficient	S.E.	t-value	P	Conclusion
H1	PSR	→	SCR	0.170	0.032	3.439	***	Supported
H2	RER	→	SCR	0.200	0.039	3.524	***	Supported
H3	PAR	→	SCR	0.209	0.047	3.316	***	Supported
H4	IRR	→	SCR	0.229	0.031	4.649	***	Supported
H5	CAR	→	SCR	0.218	0.050	3.634	***	Supported
H6	KNR	→	SCR	0.103	0.035	2.168	0.030	Supported

Note:

* P < 0.05;

** P < 0.01;

* * * P < 0.001

## 6. Summary

### 6.1. Conclusion

Based on grounded theory, this study systematizes and explores the key elements and formation mechanisms of supply chain resilience. Open coding, axial coding, selective coding, and a theoretical saturation test on the semi-structured interview data reveal that product supply resilience, resource resilience, partner resilience, information response resilience, capital resilience, and knowledge resilience are the six key elements of supply chain resilience. Among them, product supply resilience and resource resilience help to improve the absorption capability of supply chain resilience and act in the readiness phase of supply chain; partner resilience and information response resilience help to improve the adaptation capability of supply chain resilience and act in the response phase of supply chain; capital resilience and knowledge resilience help to improve the recovery capability of supply chain resilience and act in the recovery phase of supply chain; and capital resilience and knowledge resilience help to improve the recovery capacity of supply chain resilience and act in the recovery phase of supply chain. Based on these findings, a theoretical model is constructed, and corresponding hypotheses are proposed. Then, a questionnaire is designed based on the results of axial coding, and a small sample pre-survey verifies the reasonableness of the questionnaire. Finally, the structural equation modeling method is used to evaluate the theoretical model based on questionnaire data from the formal survey. The validation results indicate that product supply resilience, resource resilience, partner resilience, information response resilience, capital resilience, and knowledge resilience positively affect supply chain resilience, and the proposed hypotheses are all supported.

### 6.2. Implications

This study compensates for the lack of research in the identification of key elements of supply chain resilience, expands the study of key elements and formation mechanism of supply chain resilience, enriches the theoretical research perspective and empirical research scenarios of supply chain resilience, and is a valuable addition to the existing theoretical system of supply chain resilience, so it has some theoretical value. In the meantime, the scale of six key elements designed by this study through axial coding has been validated, providing a benchmark for designing associated questionnaires and developing the scale. In addition, the six identified key elements of supply chain resilience are the top priorities for enhancing supply chain resilience, indicating the management’s primary concerns. The categories and concepts illustrated in the coding process of the six key elements can serve as a detailed reference for practitioners seeking to enhance supply chain resiliency and have practical significance.

### 6.3. Research limitations and perspectives

This study mainly has the following limitations: (1) The six key elements of supply chain resilience identified come from the grounded theory, and their empirical data inevitably have some subjectivity, which may influence the coding results. (2) The division of the six key elements into the three phases of supply chain resilience based solely on qualitative interview data lacks a certain level of scientific validity. (3) The conclusions of this study are somewhat universal and lack industry-specific targeted research.

In the future, scholars can classify the phases of action of the six key elements from a quantitative perspective. Moreover, the key elements of supply chain resilience can be extracted based on different industries in a targeted manner. In addition, effective and efficient paths to build supply chain resilience can be proposed based on the six key elements of supply chain resilience in a targeted manner.

## Supporting information

S1 Data (XLSX)

S2 Data (XLSX)
